# School Participation Questionnaire (SPQ): Italian Translation, Cultural Adaptation, and Pilot Testing in Children with Neurodevelopmental Disorders

**DOI:** 10.3390/brainsci14070644

**Published:** 2024-06-26

**Authors:** Giorgia Pietragalla, Giovanni Galeoto, Marco Moresi, Martina Ruffini, Rachele Simeon, Francescaroberta Panuccio, Donatella Valente, Anna Berardi

**Affiliations:** 1 Department of Human Neurosciences, School of Occupational Therapy, Sapienza University of Rome, 00185 Rome, Italy; giorgiapietragalla@gmail.com; 2 Department of Human Neurosciences, Sapienza University of Rome, 00185 Rome, Italy; giovanni.galeoto@uniroma1.it (G.G.); rachele.simeon@uniroma1.it (R.S.); francescaroberta.panuccio@uniroma1.it (F.P.); donatella.valente@uniroma1.it (D.V.); 3 IRCSS Neuromed, Via Atinense, 18, 86077 Pozzilli, Italy; 4 Vaclav Vojta Cooperative Center for Neuromotor Rehabilitation, 00146 Rome, Italy; marco.moresi@centrovojta.com; 5 Center Don Gnocchi “S. Maria della Pace”, 00135 Rome, Italy; martina.ruffini@uniroma1.it

**Keywords:** school, participation, environment, disability, assessment, cultural adaptation, validation

## Abstract

Introduction: According to leading occupational therapy models, the environment appears to be a key element in fostering occupational performance and participation. There is an emerging need to identify an instrument that can assess these aspects in the school environment. Currently, there are no rating scales in Italy for the school participation of children with neurodevelopmental disorders. For this reason, this study aims to culturally adapt and translate the School Participation Questionnaire (SPQ) to Italian and to pilot test the translated Italian version on children with neurodevelopmental disorders. Methods: The original scale was translated from English to Italian using the guidelines “Translation and Cultural Adaptation of Patient Reported Outcomes Measures—Principles of Good Practice”. The psychometric properties analyzed were the following: content validity, construct validity, cross-cultural validity, and internal consistency. Construct validity was assessed using the Italian version of the Sensory Processing Measure (SPM). Results: The Italian version of the SPQ was administered to 22 children. The mean ± SD of the score was 9.32 ± 1.36; Cronbach’s α was 0.935 (*p* < 0.01). The Pearson’s correlation coefficient with the SPM scores was −0.622 (*p* < 0.01). Conclusions: This pilot testing study for the validation of the Italian version of the SPQ showed good validity and reliability results. Thanks to this study, it would be possible to further analyze the tool for the evaluation of the environment and school participation of children with neurodevelopmental disorders, in particular autism spectrum disorder. It is a quick and easy instrument that focuses on the environment as an active variable in the child’s occupational performance.

## 1. Introduction

The World Health Organization (WHO), within the “International Classification of Functioning, Disability and Health—ICF”, defines participation as “the involvement of a person in a life situation” [1]. Keeping this definition in mind, it is possible to introduce a specific form of participation: scholastic participation. According to some authors [2,3], the definition cited above is not complete, as school participation should also be understood in terms of the attendance and involvement of the child. This refers to their activities, how often they are carried out, and their interest in them. Based on these statements, the motivations that led to the present study will follow [4,5].

Remembering that education is a fundamental human right [6], it is appropriate to consider school as one of the environments most frequented by children, which contributes to their development both on a social and emotional level and encourages participation. Furthermore, it is possible to state that the latter is fundamental in the school environment for developing new skills, expressing creativity and personal identity, and establishing significant relationships with the educator and peers.

From some studies in the literature [3,7,8,9], it emerges that at school, children with special educational needs (SEN) experience lower and limited levels of participation compared to their peers who do not present any obvious disability. This is due to various factors, among which mainly environmental ones emerge.

The environment, in general, can be divided into four different levels: micro, meso, exo, and macroenvironment [3]. Specifically, the microenvironment is closest to the child and includes the relationships, interactions, and structures in the immediate vicinity; therefore, the environmental level directly influencing the child’s participation in the school environment is defined.

It is possible to propose a further subdivision of the school context into the physical environment (for example, classrooms, furnishings, teaching materials, etc.) and social environment (for example, peers, teachers, parents, etc.), remembering that each element that makes up this environment influences the behavior and actions produced by the child [10]. This idea that a child’s learning and development are favored by the interaction between the subject and a favorable environment dates back to the time of the psychologist Jean Piaget and was then carried forward and confirmed by scientific evidence demonstrating the influence of the environment on participation and the general functioning of the individual [3,11,12].

In fact, the International Classification of Functioning, Disability and Health—ICF [1] defines disability as “the consequence or result of a complex relationship between an individual’s health condition, personal factors, and environmental factors”; therefore, it can be stated that an environment rich in stimuli causes children to naturally express their personality and, at the same time, acquire new skills. Correspondingly, an unstimulating environment can limit the learning and social participation of some children, generating important limitations in their global functioning.

In a study conducted in 2019 by Donald Maciver [11], the four areas that seem to influence school participation in a child and on which it is necessary to focus have been identified: identity, competence, the experience of the mind and body, and the environment. Identity includes all the mechanisms linked to the child’s “Being”, their thoughts and feelings towards themself, their preferences, self-perceptions, significance, internalization and perceptions of roles, and internalization of habits and routine.

Competence includes all the mechanisms related to “Doing”, the behavioral aspects, and how children participate. These mechanisms include making choices, persistence, meeting role expectations, satisfying routine habits and expectations, organization and planning, motor skills, and communication skills.

On the other hand, the experience of mind and body refers to the symptoms associated with disability, which determine the effects on participation. These may include pain, fatigue, anxiety, and mood.

Finally, the environment is seen as the set of contexts that can facilitate or not facilitate school participation and is divided into five related areas: the structures and organization of the school (adaptation to the child, flexibility, routine), adults (creators of opportunities, attitudes, knowledge and skills, practical structures), peers (support, friendship, attitudes), physical spaces (available/accessible, suitable), and objects (available/accessible, suitable), which summarize and concretize the abovementioned concept of the physical and social environment. Conducting assessments of participation in the child’s natural environment, such as the classroom, is beneficial [13]. However, there are several general barriers to using measures in schools. These include demands on time [14], training, or knowledge [15], and whether a particular measure has face validity [16]. Poor returns for data collection efforts are common, and teachers may express concerns about measures not being necessary or relevant.

Considering what has been said previously, the need to identify a tool that evaluates the factors that influence school participation in Italy and that allows the occupational therapist to analyze both the physical and social environment is increasingly urgent. For this reason, the authors selected the School Participation Questionnaire (SPQ), which was originally taken in collaboration with teachers, school leadership, pediatric occupational therapists, and parents [12,17,18,19].

The SPQ is an assessment tool that analyzes the school environment surrounding the child, evaluating how more or less inclusive it is. This study aims to translate and culturally adapt the SPQ and pilot test the Italian version on children with neurodevelopmental disorders (NDDs); in particular, the hypothesis testing is that school participation in children with NDDs is related to their sensory profile.

## 2. Methods

This study was conducted by a research group at Sapienza University, Rome, who were involved in different studies on rehabilitation and the validation of outcome measures.

### 2.1. School Participation Questionnaire (SPQ)

The SPQ is a questionnaire created to support the identification of children with reduced and/or limited participation in the school context who may need additional support, in particular children with autism spectrum disorder. It is identified as one of the tools to support inclusion and participation. Teachers can use it to understand the needs of individual children, and school management can use it to understand the range of needs and problems of the various groups/schools. It is, therefore, designed to ensure a rapid understanding of children and environments and to facilitate timely support that adapts to each child’s needs [18,19].

Before the creation of the SPQ by Donald Maciver, no tool allowed an evaluation of constructs related to participation in schools [19], and this, in addition to being an important tool for teachers, could also become an evaluation tool for specialists who follow children with disabilities, such as occupational therapists, in such a way as to be able to have a global vision of the child and also be able to intervene in improving the school environment. The SPQ aims to systematically address and analyze the four key factors that influence a child’s social participation:

- Being represents the set of thoughts and feelings that the child feels about themself, understanding their abilities, understanding their role, and feeling like a member of the school community.
- Doing represents what the child does at school, for example, following the rules and showing interest in activities.
- Symptoms are everything that children with disabilities experience daily in a school environment, for example, pain, anxiety, and tiredness.
- Environment represents the physical and social characteristics of the school context.

Specific items correspond to these four areas: Being has 9 items; Doing has 11 items; Symptoms has 5 items; and Environment has 19 items.

Participants must indicate to what extent they agree with each of the 44 items by assigning a score from 1 to 4 to each of them (1 = Disagree; 2 = Somewhat Disagree; 3 = Somewhat Agree; 4 = Agree); if participants are not sure which score to assign between two, it is always best to choose the lower one.

A total score was not included in the original version of the scale. However, based on the trend of the scores of the individual items within each area, the ones that require greater intervention are identified. In adapting the Italian version, however, it was deemed necessary to outline the raw scores of the different areas, calculated as the sum of the scores of the individual items in each area, to monitor the statistical progress of the conducted study more easily.

The estimated administration time is approximately 10 min; completing it all in one go is unnecessary as it can also be completed one section at a time.

The time frame taken into consideration when filling out the questionnaire refers to the child’s behavior during the previous two weeks. It is important to consider the child’s attitudes generally, not just their positive or negative days. Furthermore, the SPQ focuses on the school, so when it asks about activities carried out, roles held, etc., only those that take place at school should be considered.

In this work, the first administration of the SPQ will also be associated with the administration of the “Sensory Processing Measure—SPM” rating scale to evaluate the construct validity mentioned above.

The SPM [20] is a set of assessment protocols that allows you to measure sensory processing difficulties, practices, and participation in school-age children between 6 and 12 years old. It is based on Ayres’ theory of sensory integration, according to which sensory processing and the integration of sensory input constitute a critical neurobehavioral process that strongly influences child development. The theory holds that a child with impaired sensory processing may not be able to learn functionally or perform activities of daily living adequately. Sensory processing difficulties often impair higher-level integrative functions, such as social participation and praxis (the ability to plan and organize movement).

The version adapted into Italian consists of two protocols:

- SPM Home: composed of 75 items filled in by the parent or person caring for the child.
- SPM School: composed of 62 items and is filled in by the child’s main teacher or by a teacher’s assistant who knows the child well; in any case, the evaluator must observe the child in class for at least 1 month to complete the form.

Each protocol requires 15 to 20 min to be completed by an evaluator and an additional 5 to 10 min to be evaluated by an occupational therapist or another evaluator.

Each item is assigned a qualitative score, varying between Never, Occasionally, Frequently, and Always. Each temporal adverb is then assigned a quantitative score from 1 to 4, based on the different areas; finally, the scores are inserted into a calculation grid to define the child’s sensory profile, which is classified as Typical (score from 40 to 59), Some Difficulties (score from 60 to 69), or Definite Dysfunction (score from 70 to 80).

Both protocols, Home and School, provide 8 normative reference standard scores:

- Social participation (SOC): home module, 10 items; school module, 10 items.
- View (VIS): home module, 11 items; school module, 7 items.
- Hearing (UDI): home module, 8 items; school module, 7 items.
- Tact (TAT): home module, 11 items; school module, 8 items.
- Body awareness (COR): home module, 10 items; school module, 7 items.
- Balance and movement (EQU): home module, 11 items; school module, 9 items.
- Planning and ideas (IDE): home module, 9 items; school module, 10 items.
- Total raw score (TOT): home module, 56; school module, 42.

The two protocols were designed to be used together, thus providing a comprehensive overview of the child’s sensory functioning. However, if you only have access to one environment, you can use them individually.

The SPM is intended to facilitate the identification and treatment of children with sensory processing difficulties; it was developed by occupational therapists, but the information provided will also be useful to other professionals [20].

### 2.2. Translation and Cultural Adaptation

This study was initiated in compliance with the 2005 “Translation and Cultural Adaptation of Patient Reported Outcomes Measures—Principles of Good Practice Guidelines” [21], in which a review is made of the 12 major guidelines available in the literature for translation and cultural adaptation. In addition, the COnsensus-based Standards for the selection of health Measurement INstruments (COSMIN) methodology was followed [21,22,23].

After receiving permission from the developers of the original tool, the first stage in the translation process was forward translation. The original English version of the tool was translated to Italian by a panel of two native speakers of the target language; one of them was an occupational therapist expert in the evaluation of the environment and school participation of children with neurodevelopmental disorders, while the other forward translator was naïve in the disease involved and the construct measured by the tool. These individuals produced three independent translations. An independent native speaker of the target language who had not been involved in any of the forward translations synthesized the results of the translations.

Working from the literally translated temporary version of the questionnaire, two native English speakers, naïve about the disease involved and the construct to be measured, independently translated the questionnaire and performed the backward translation without having seen the original version. The backward-translated version of the instrument was compared with the original one, and any discrepancies between the two English versions were solved by a third independent reviewer who was not involved in any of the translations. This version was submitted to the developers of the instrument for their approval. After this step, a feedback report of the translations was written.

For the cultural adaptation process (content validity), in order to adapt the translated version to Italian culture, two Italian rehabilitation professionals (occupational therapists), one clinical psychologist, and a person of the target population (a teacher), who were familiar with both English and Italian, reviewed the first translated version and then reworded and reformulated some items to ensure relevance, comprehensiveness, and comprehensibility. The expert committee’s role was to consolidate all the versions of the questionnaire and to develop what would be considered the pre-final version of the questionnaire for field testing from the perspective of professionals and the teacher.

### 2.3. Pilot Test—Population

This pre-final translated version of the instrument was preliminarily applied to a sample of the target population (teachers of children with neurodevelopmental disorders [NDDs]). In line with the original version of the tool and the COSMIN criteria international guideline [23], the sample size required for a pilot test was 20 teachers (1/3 of the population administered in the original validation study) [24], and the inclusion criteria were as follows:

- aged between 6 and 12 years;
- attend primary school at the time of recruitment;
- present a form of disability attributable to the category of NDD.

Children out of this age range and without a diagnosis of a disability attributable to the category of NDD were excluded from the study.

All the people involved in the study, parents and their teachers, were informed about the purposes of the test, its duration, and the privacy protection procedures. Parents were informed in order to obtain their consent for the teacher to evaluate their children, while teachers were informed because they were the people in charge of filling out the assessment tool. Only subjects whose informed consent was completed and signed were included in the study [19].

### 2.4. Pilot Test—Psychometric Properties

Content validity measures the degree to which the content of the tool is an adequate reflection of the content of the construct to be measured. This psychometric property was assessed by asking the twenty included teachers and ten experienced occupational therapists (with at least 5 years of experience in clinical settings with children) if, based on their professional experience with school participation in children with NDD, each item (instructions, item, response options, and recall period) was relevant and comprehensive. The included participants had to assign a value of 0 (not adequate) or 1 (adequate) for relevance, comprehensiveness, and comprehensibility.

Construct validity defines the degree to which the scores of a tool are consistent with the hypothesis based on the assumption that the instrument validly measures the construct to be measured. The hypothesis testing was that school participation in children with NDD is related to their sensory profile. For this reason, construct validity was analyzed by comparing the scores obtained at the SPQ with scores obtained at the SPM through Pearson’s correlation coefficient. When interpreting the results, the following ranges were considered: r > 0.70, a strong correlation; 0.50 < r < 0.70, a moderate correlation; and r < 0.50, a weak correlation. The significance level was set as a *p*-value less than or equal to 0.05. Cross-cultural validity/measurement invariance refers to the possibility of applying a measurement instrument initially generated in a single culture to another culture different from the original one in an equivalent way . This property aims to investigate whether the items of a tool behave similarly in different populations. For this study, gender, age, and diagnosis were considered. Mean scores and standard deviations were calculated. Moreover, box plots showing graphical distributions of scores were generated.

Internal consistency describes the degree to which the scale items are related; it is the ability to measure a single concept/characteristic or domain/dimension of a concept with minimal error, and this property is mainly estimated through Cronbach’s alpha coefficient. The latter measures the global correlation between the items within a scale; an alpha coefficient of 0.70 is considered the minimum acceptable level of internal consistency.

Statistical analysis will be performed using IBM-SPSS version 23.00 software.

## 3. Results

### 3.1. Translation and Cultural Adaptation

The translation and cultural adaptation process of the School Participation Questionnaire (SPQ) were conducted following international guidelines [12,18,19] and with the supervision of the original authors and a panel of experts, who ensured that the original meaning of the items was maintained. The source of distortion were some expressions used in the original version which, translated into the adaptation language, would not have had an explanatory meaning of the real concept intended to be expressed.

Some examples are the following:

- The child is free from anxiety in school—The child is free from anxiety in school;
- The child seems well-slept when they arrive for school—The child seems to have slept well when he arrives at school;
- The child is pain-free during school—The child is pain-free during school;
- Visual supports are in place (e.g., timetable, classroom labels)—Visual supports are present (e.g., timetables, classroom labels).

An external judge, not involved in any of the translations, analyzed and compared the three translations to combine them. Through this process, a translated version was created in which the expressions mentioned above were translated as follows:

- The child does not show anxiety at school;
- The child appears to be rested when he arrives at school;
- The child has no pain during school;
- There are visual supports (e.g., timetables, labels for identifying objects).

The translated version of the instrument is reported in Appendix A.

### 3.2. Pilot Test—Population

The population was recruited starting from 6 June 2023 through dissemination within some schools in Rome. After a thorough evaluation, in accordance with the inclusion criteria, 22 teachers of children with neurodevelopmental disorders (NDDs) agreed to participate in the study. All the people involved in the study, i.e., parents and their teachers, were informed about the purposes of the questionnaire, the duration of the study and the privacy protection procedures. Only subjects whose informed consent was completed and signed were included in the study. From 6 June 2023 to 10 October 2023, 22 teachers of children of an average age of 9.32 ± 1.36 years were assessed; the administration took place through a paper compilation of the SPQ and SPM questionnaires by the support teacher or support person of the child. For each participant in the study, demographic data, such as gender, diagnosis, class, and presence or absence of a support teacher, were considered. These characteristics are reported in Table 1.

### 3.3. Pilot Test—Psychometric Properties

Content validity: The twenty-two teachers included and the ten occupational therapists with experience in school participation of children with NDDs evaluated the relevance, comprehensiveness, and comprehensibility of the instructions, items, and response options of the SPQ. All people involved evaluated the tool and considered it adequate (score 1).

Construct validity: To evaluate the hypothesis testing, the average scores obtained on the subscales of SPQ were compared using the Pearson’s correlation index with the Sensory Profile Measure (SPM). This analysis allowed authors to compare the correlation between the constructs represented by the tools. As can be seen from the results reported in Table 2, the data with * are statistically significant as *p* < 0.05, while the data with ** are highly significant as *p* < 0.01.

In particular, it was found that the SPQ-TO BE subscale has a statistically significant correlation with the SPM-SOC (social participation) subscale with * *p* < 0.05 and appears to have a higher level of significance with ** *p* < 0.01 with the subscales SPM-UDI (hearing), SPM 33–36 (taste), SPM-COR (body awareness), SPM-IDE (idea planning) and with the total of the SPM.

The SPQ-TO DO subscale has a statistically significant correlation with the SPM-SOC (social participation) and SPM-VIS (vision) subscales with * *p* < 0.05 and appears to have a higher level of significance with ** *p* < 0.01 with the subscales SPM-UDI (hearing), SPM 33–36 (taste), SPM-COR (body awareness), SPM-IDE (idea planning) and with the SPM total. The SPQ-SYMPTOMS subscale has a statistically significant correlation with the SPM-UDI (hearing), SPM-COR (core awareness), and SPM-EQU (balance and movement) subscales with * *p* < 0.05; it appears to have a higher level of significance with ** *p* < 0.01 with the SPM-VIS (sight) and SPM-IDE (idea planning) subscales and with the SPM total.

**Figure 1:**
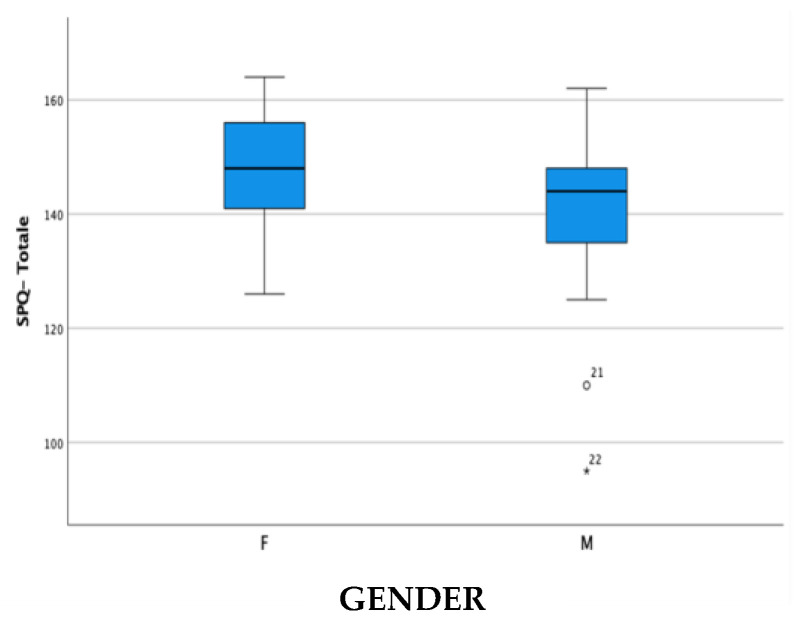
Score difference in the School Participation Questionnaire in Females (F) and Males (M).

**Figure 2:**
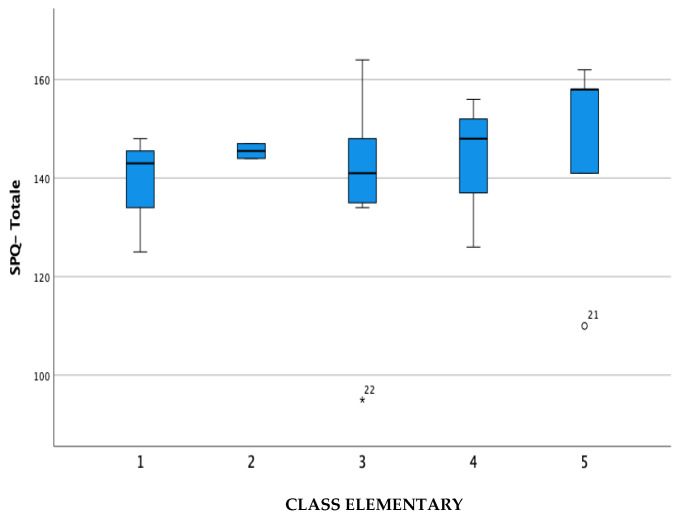
Score difference in the School Participation Questionnaire in children at first, second, third, fourth, and fifth year of elementary school.

**Figure 3:**
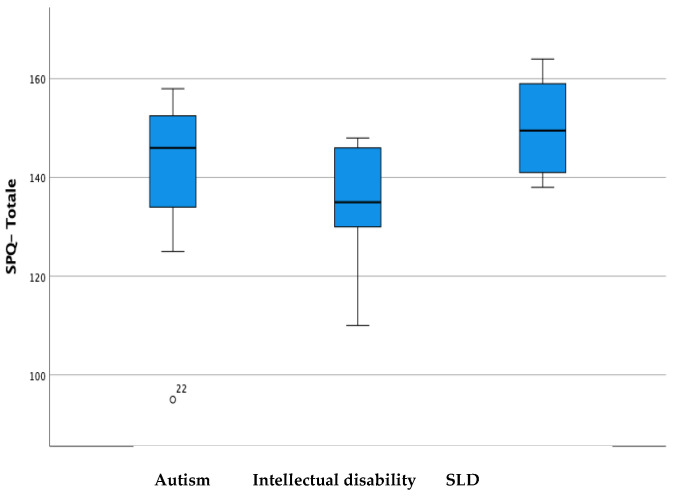
Score difference in the School Participation Questionnaire in children with autism spectrum disorder, intellectual disability or Specific Learning Disorder (SLD).

For cross-cultural validity, Table 3, Table 4 and Table 5 show the differences in the mean scores of the subpopulations relating to gender, class, and diagnosis. Subsequently, the data were represented in the boxplots in Figure 1, Figure 2 and Figure 3.

Internal consistency: For all items, the four subscales, and the total of the assessment instrument, the entire consistency was analyzed through Cronbach’s alpha analysis. This analysis allows authors to state that the same instrument’s subscales contribute coherently to analyzing the same construct (difficulty with environmental barriers, social integration, and participation). An analysis was also conducted to evaluate how the presence or absence of each item of the four subscales influences the total alpha value for that specific subscale. From the data reported in Table 6, all the SPQ subscales have an excellent Cronbach’s alpha value (α > 0.7).

## 4. Discussion

The objective of the present study was to translate and culturally adapt the School Participation Questionnaire (SPQ) and pilot test the Italian version in children with neurodevelopmental disorders (NDDs). The translation and cultural adaptation were conducted by applying international guidelines [21,22,23,25], guaranteeing, with the supervision of a panel of experts, the maintenance of the original meaning of the items and adequate content validity.

For construct validity analysis, a comparison was carried out with the SPM scale, as the it investigates the child’s sensory processing, practices, and social participation, focusing not only on the home context but also reserving a suitable module to investigate these aspects within the school environment.

For this investigation, Pearson’s coefficient was used, which showed an excellent correlation between the SPQ total and the SPM total and a good correlation between most of the subscales of both instruments, except for the SPM-TAT subscale which investigates the tactile sense and the SPM-EQU subscale which investigates the child’s balance and movement, aspects not present in our questionnaire and therefore unrelated. Specifically, the SPQ-ENVIRONMENT subscale presented a significant correlation with both the SPM-IDE subscale (planning of ideas), since both investigate the area of planning by analyzing two primary components of the child’s occupation: routines and organizational activities, both with the SPM-UDI subscale (hearing), as in the classroom environment, there are often available spaces suitable for responding to the personal needs of a child who, for example, concerning the SPM, seems to have hearing difficulties and therefore can effectively use these spaces in order to improve their self-regulation with respect to strong external auditory stimuli.

Regarding cross-cultural validity, a specific analysis concerning gender, class, and diagnosis was conducted to investigate the trend of the measurements carried out using the SPQ’s four subscales. 

The data express a slight gender difference, especially in the SPQ-TO DO and SPQ-SYMPTOMS subscales, and a level of adaptation and participation that tends to increase in relation to the progression of the classes, except for the SPQ-TO DO subscale, which remains generally limited despite the growth of the child mainly due to personal limitations inherent to the present disability. Finally, in line with what was expected, notable differences were found in relation to the type of diagnosis, and it was found that children diagnosed with ASD had higher levels of participation compared to children with autism spectrum syndrome and children with intellectual disability and that the latter have greater limitations than the other two.

In support of the aforementioned, it is important to emphasize that on many occasions, children diagnosed with autism spectrum disorder, especially if cognitively performing, implement functional adaptation strategies to be able to maintain adequate levels of social participation. The importance and innovation brought by the SPQ consist of placing the analysis and evaluation of the environment at the center rather than the abilities and type of behavior implemented by the child within the environment itself, aspects which are recorded from the SPM. Therefore, considering these statements, the active performance variable is represented by the environment and not by the child, as the latter has specific characteristics that are influenced positively or otherwise by the context in which they live. Therefore, the intervention must be mainly focused on environmental changes to improve occupational performance rather than on the subject’s characteristics. The reliability assessment was conducted using Cronbach’s alpha coefficient; thanks to this analysis, it was possible to demonstrate that both the entire scale and the four subscales present a high internal consistency with an alpha score of 0.935 (total SPQ) and 0.892, 0.857, 0.766, 0.856 (total subscales). These results are consistent with the original validation study, which presents Cronbach’s alpha values of 0.89, 0.94, 0.0.84, and 0.94 (total subscales). In the literature, the SPQ was validated by teachers of children (4.10–12 years old) with additional support needs, with a range of disabilities including intellectual disability, autism spectrum disorder, and learning difficulties (special education needs) [1,2], and typical development. Psychometric properties which have been analyzed were the following: construct validity with Participation and Environment Measure for Children and Youth (PEM-CY), test-retest (ICCs = 0.64, 0.61, 0.78, 0.6), interrater reliability (ICCs = 0.85, 0.71, 0.90, 0.), and internal consistency (alphas = 0.89, 0.9, 0.94, 0.7) [1]. Content validity index k > 0.74; Cronbach’s alpha > 0.80 [1]. Salas et al. [3] does not assess any psychometric properties, but correlates two populations (children with typical development and children with special educational needs).

Therefore, it is possible to state that the results obtained highlight a notable correlation between the different items of the scale and indicate that the measurements obtained from the Italian version of the assessment tool are consistent and reliable.

Furthermore, it is possible to state that, thanks to the analysis conducted to record the variations in the alpha value as the items vary, the presence of each single item is important to maintain high alpha levels. Therefore, each item is essential for the reliability of the instrument.

### Limitations

The study only conducted preliminary testing on 22 children, which limits the generalizability and applicability of the results. However, the aim of the study was to pre-test the tool to evaluate its applicability to a larger population; in addition, the sample size is considered adequate for pre-testing according to the COSMIN checklist. This study represents the pilot test phase of the validation study; further analysis should validate the instrument on a larger sample size, also considering the healthy population. Regarding the SPQ-ENVIRONMENT subscale, the results demonstrated excessively high scores compared to the child’s sensory perception found in the SPM. This proves to be a limitation of the SPQ scale, as the compilation takes place exclusively by school staff, and there needs to be a comparison with the child’s or parent’s perception. Therefore, the results of the SPQ-ENVIRONMENT subscale could be influenced by the teacher’s perception and, therefore, present a distorted result. It is recommended to further validate the environmental subscale and consider incorporating the perspectives of children and parents to obtain more comprehensive data. Finally, it was not possible to compare the SPQ with another participation questionnaire such as the Participation and Environment Measure Children and Youth (PEM-CY) because such tools are not yet available in Italian. Further studies should consider validating other correlated measurement tools.

## 5. Conclusions

In conclusion, it is possible to state that the culturally adapted Italian version of the School Participation Questionnaire (SPQ) has proven to be ready to be validated, demonstrating good values of initial validity and reliability for evaluating constructs relating to school participation in children with neurodevelopmental disorders.

Therefore, this study has allowed for the creation of a new measurement and evaluation tool suitable for professionals, such as occupational therapists and teachers, to both fully understand the child’s difficulties and limitations and be able to collaborate on the creation of a specific intervention in relation to their abilities. In particular, the benefit to the mental health of children with neurodevelopmental disabilities, particularly ASD, is clear when thinking from a holistic well-being perspective, considering the environment as a fundamental element of an individual’s health, as recommended by the International Classification of Functioning, Disability, and Health (ICF). In addition to what has been said and from a future perspective, the validation of the SPQ could also be carried out on a population of typically developed children to be able to analyze when and in relation to which factors they begin to demonstrate difficulties in social and scholastic participation; this supports the fact that oftentimes, poor inclusiveness is not necessarily dependent on the presence of disabilities.

## Figures and Tables

**Table 1:** Demographic characteristic of the included participants.

		Frequency (%)
Gender	Female	5 (22.7)
	Male	17 (77.3)
Diagnosis	Autism	7 (31.8)
	Intellectual disability	7 (31.8)
	Specific Learning Disorders	8 (36.4)
Support teacher		17 (77.3)
Class	First	3 (13.6)
	Second	2 (9.1)
	Third	9 (40.9)
	Fourth	3 (13.6)
	Fifth	5 (22.7)

**Table 2:** Construct validity between assessment tools: Pearson correlation index.

		SPM SOC	SPM VIS	SPM UDI	SPM TAT	SPM 33–36	SPM COR	SPM EQU	SPM IDE	SPM TOTALE
SPQ-TO BE	Pearson	−0.446 *	−0.401	−0.657 **	−0.081	−0.548 **	−0.672 **	−0.352	−0.712 **	−0.571 **
	Sign	0.037	0.065	0.001	0.720	0.008	0.001	0.108	0.000	0.006
SPQ-TO DO	Pearson	−0.504 *	−0.532 *	−0.731 **	−0.205	−0.625 **	−0.607 **	−0.376	−0.818 **	−0.640 **
	Sign	0.017	0.011	0.000	0.360	0.002	0.003	0.085	0.000	0.001
SPQ-SYMPTOMS	Pearson	−0.033	−0.622 **	−0.490 *	−0.343	−0.348	−0.498 *	−0.535 *	−0.541 **	−0.609 **
	Sign	0.883	0.002	0.021	0.118	0.112	0.018	0.010	0.009	0.003
SPQ-ENVIRO	Pearson	−0.104	−0.358	−0.431 *	0.119	−0.424 *	−0.341	−0.197	−0.679 **	−0.322
	Sign	0.646	0.102	0.045	0.597	0.050	0.120	0.380	0.001	0.144
SPQ-Total	Pearson	−0.350	−0.553 **	−0.702 **	−0.105	−0.607 **	−0.628 **	−0.404	−0.858 **	−0.622 **
	Sign	0.110	0.008	0.000	0.641	0.003	0.002	0.062	0.000	0.002

* *p* < 0.05; ** *p* < 0.01. Social participation (SOC), vision (VIS), hearing (HEA), touch (TOU), body awareness (BOD), balance and motion (BAL), planning and ideas (PLA), total sensory systems (TOT).

**Table 3:** Cross-cultural validity: differences between males and females in the mean scores of the assessment tool.

	Gender	Mean (Dev.st)
SPQ-TO BE	Female	30.2 (3.6)
	Male	28.29 (5.0)
SPQ-TO DO	Female	32.6 (6.3)
	Male	29.8 (5.6)
SPQ-SYMPTOMS	Female	15.4 (3)
	Male	15.1 (3)
SPQ-ENVIRONMENT	Female	68.8 (4.3)
	Male	67 (7.3)
SPQ-Total	Female	147 (14.6)
	Male	140.2 (17.2)

**Table 4:** Cross-cultural validity: differences in the average scores of the assessment tool in different elementary classes.

	Class	Mean (Dev.st)
SPQ-TO BE	1	26.4 (5.1)
	2	31.00
	3	28 (4.8)
	4	28.7 (4)
	5	30.6 (6.1)
SPQ-TO DO	1	31 (4.3)
	2	29.5 (2.1)
	3	30.2 (7.6)
	4	30.7 (5)
	5	30.8 (5.7)
SPQ-SYMPTOMS	1	16.6 (1.2)
	2	17 (2.8)
	3	14.4 (2.9)
	4	14.7 (3.1)
	5	15 (3.8)
SPQ-ENVIRONMENT	1	64.7 (9)
	2	68 (2.8)
	3	66.4 (7.7)
	4	69.3 (5.7)
	5	69.4 (6.8)
SPQ-Total	1	138.7 (12.1)
	2	145.5 (2.1)
	3	139.1 (19)
	4	143.3 (15.5)
	5	145.8 (21.6)

**Table 5:** Cross-cultural validity: differences in the different types of diagnoses in the average scores of the two assessment tools.

	Diagnosis	Mean (Dev.st)
SPQ-TO BE	Autism	27.7 (5.3)
	Intellectual disability	26.7 (4.6)
	specific learning disorder	31.4 (3.3)
SPQ-TO DO	Autism	28.4 (67)
	intellectual disability	28.4 (4.4)
	specific learning disorder	34 (4.7)
SPQ- SYMPTOMS	Autism	15.9 (2.3)
	intellectual disability	14.4 (3)
	specific learning disorder	15.1 (3.3)
SPQ- ENVIRONMENT	Autism	66.9 (10.1)
	intellectual disability	65.4 (4.3)
	specific learning disorder	69.6 (4)
SPQ-Total	Autism	138.8 (22.3)
	intellectual disability	135 (13.7)
	specific learning disorder	150.1 (9.9)

**Table 6:** Internal consistency of rating scales: Cronbach’s alpha analysis.

Items	Cronbach’s Alpha If Item Is Deleted-Subscale	Subscale Total Alpha	Cronbach’s Alpha If Item Is Deleted-Scale Total	Alpha Total Scale
SPQ-TO BE item 1	0.877	0.892	0.932	0.935
SPQ-TO BE item 2	0.889		0.934	
SPQ-TO BE item 3	0.901		0.934	
SPQ-TO BE item 4	0.882		0.933	
SPQ-TO BE item 5	0.873		0.933	
SPQ-TO BE item 6	0.873		0.932	
SPQ-TO BE item 7	0.878		0.933	
SPQ-TO BE item 8	0.864		0.932	
SPQ-TO BE item 9	0.878		0.932	
SPQ-TO DO item 1	0.854	0.857	0.933	
SPQ-TO DO item 2	0.841		0.933	
SPQ-TO DO item 3	0.842		0.933	
SPQ-TO DO item 4	0.824		0.931	
SPQ-TO DO item 5	0.832		0.932	
SPQ-TO DO item 6	0.847		0.933	
SPQ-TO DO item 7	0.847		0.933	
SPQ-TO DO item 8	0.831		0.931	
SPQ-TO DO item 9	0.868		0.936	
SPQ-TO DO item 10	0.839		0.933	
SPQ-TO DO item 11	0.858		0.933	
SPQ-SYMPTOMS item 1	0.736	0.766	0.935	
SPQ-SYMPTOMS item 2	0.751		0.937	
SPQ-SYMPTOMS item 3	0.744		0.933	
SPQ-SYMPTOMS item 4	0.679		0.935	
SPQ-SYMPTOMS item 5	0.759		0.931	
SPQ-ENVIRONMENT item 1	0.853	0.856	0.935	
SPQ-ENVIRONMENT item 2	0.852		0.935	
SPQ-ENVIRONMENT item 3	0.843		0.933	
SPQ-ENVIRONMENT item 4	0.854		0.935	
SPQ-ENVIRONMENT item 5	0.849		0.933	
SPQ-ENVIRONMENT item 6	0.843		0.933	
SPQ-ENVIRONMENT item 7	0.852		0.934	
SPQ-ENVIRONMENT item 8	0.852		0.936	
SPQ-ENVIRONMENT item 9	0.842		0.933	
SPQ-ENVIRONMENT item 10	0.849		0.934	
SPQ-ENVIRONMENT item 11	0.856		0.934	
SPQ-ENVIRONMENT item 12	0.845		0.932	
SPQ-ENVIRONMENT item 13	0.844		0.934	
SPQ-ENVIRONMENT item 14	0.868		0.936	
SPQ-ENVIRONMENT item 15	0.852		0.935	
SPQ-ENVIRONMENT item 16	0.836		0.931	
SPQ-ENVIRONMENT item 17	0.849		0.934	
SPQ-ENVIRONMENT item 18	0.850		0.934	
SPQ-ENVIRONMENT item 19	0.841		0.932	

## Data Availability

The data supporting this study’s findings are available from the corresponding author upon reasonable request. The data are not publicly available due to restrictions e.g., their containing information that could compromise the privacy of research participants.

## Appendix A

Italian translated and culturally adapted version of the School Participation Questionnaire (SPQ).
